# Community-acquired *Pseudomonas aeruginosa* sepsis presenting with ecthyma gangrenosum in a neutropenic infant: a case report

**DOI:** 10.3389/fped.2025.1709166

**Published:** 2025-11-27

**Authors:** Xing Wang, Yongmei Xiao, Ting Ge, Ting Zhang, Xiaolu Li

**Affiliations:** 1 Department of Gastroenterology, Hepatology and Nutrition, Shanghai Children’s Hospital, Shanghai Jiao Tong University, Shanghai, China; 2 Institue of Pediatric Infection, Immunity and Critical Care Medicine, Shanghai Children’s Hospital, Shanghai Jiao Tong University School of Medicine, Shanghai, China

**Keywords:** neutropenia, ecthyma gangrenosum, *pseudomonas aeruginosa*, community-acquired infection, pediatric sepsis

## Abstract

**Background:**

Ecthyma gangrenosum (EG) is a distinctive cutaneous marker of *Pseudomonas aeruginosa* (*P. aeruginosa*) bacteremia, particularly in neutropenic hosts. In infants, community-acquired *P. aeruginosa* infection can precipitate fulminant septic shock with high mortality, underscoring the importance of early EG recognition.

**Case presentation:**

We report a 4-month-old ex-preterm girl presenting with fever, lethargy, and feeding intolerance. Investigations revealed profound neutropenia (ANC 0.08 × 10^9^/L), markedly elevated inflammatory markers, and a violaceous perianal plaque. Within 24 h, the lesion progressed to necrotic ulceration with septic shock. Blood and wound cultures confirmed *P. aeruginosa*. Management included meropenem therapy, continuous renal replacement for cytokine storm, intravenous immunoglobulin, and urgent surgical debridement, resulting in clinical stabilization and wound improvement.

**Conclusions:**

EG is an early sentinel sign of community-acquired *P. aeruginosa* bacteremia in neutropenic infants. Careful perianal and genital examination is essential in febrile neutropenia, and prompt initiation of anti-pseudomonal therapy with timely source control is critical to reduce mortality.

## Background

*Pseudomonas aeruginosa* (*P. aeruginosa*) is a ubiquitous Gram-negative opportunistic pathogen with remarkable environmental adaptability. Although harmless to healthy individuals, it poses a major threat to immunocompromised hosts due to its intrinsic and acquired antimicrobial resistance (1, 2). Clinically, *P. aeruginosa* is implicated in a broad spectrum of infections, including pneumonia, urinary tract infections, bacteremia, and necrotizing skin lesions, and it is also the predominant pathogen in burn wound sepsis, hot-tub folliculitis, and otitis externa. In addition, it represents a leading cause of healthcare-associated infections through colonization of indwelling medical devices, resulting in ventilator-associated pneumonia and catheter-related bloodstream infections. In patients with cystic fibrosis, it remains the most common cause of chronic pulmonary infection, with prevalence exceeding 80% in adults (2, 3).

Neutropenia, defined as an absolute neutrophil count (ANC) below 1.5 × 10^9^/L, is a critical predisposing factor for invasive bacterial infection. It is categorized as mild (1,000–1,500/*μ*L), moderate (500–1,000/μL), severe (<500/μL), and very severe (<200/μL). In infants, the physiological nadir of neutrophils occurs around the fourth week of life, with a lower reference limit of 1,000/μL, creating a vulnerable window for fulminant sepsis (4, 5). This profound neutropenia creates a permissive environment for fulminant bacterial sepsis.

Ecthyma gangrenosum (EG) is a distinctive cutaneous lesion that serves as a critical marker of *P. aeruginosa* infection, particularly in immunocompromised hosts. It is important to recognize that EG can manifest in both bacteremic and non-bacteremic forms (6); the latter typically stems from a primary localized skin inoculation. The rapid and life-threatening course of pseudomonal sepsis in susceptible individuals, such as neutropenic infants, highlights the vital importance of its early recognition. Accurate diagnosis hinges on distinguishing EG from other necrotizing skin disorders, notably pyoderma gangrenosum (PG) and necrotizing fasciitis (7). PG is a rare, non-infectious neutrophilic dermatosis with multifactorial origins—including autoimmune, inflammatory, and paraneoplastic conditions—and is characterized by painful, rapidly enlarging ulcers with violaceous, undermined borders. In contrast, necrotizing fasciitis, a life-threatening soft-tissue infection often caused by toxin-producing bacteria such as group A streptococcus, progresses rapidly to fascia necrosis and is distinguished by severe pain disproportionate to physical findings (8). It is rapidly fatal without prompt, aggressive surgical debridement, where time to intervention is the most critical variable influencing mortality. Thus, accurate differentiation among these entities based on clinical features, pain characteristics, and pathogenesis is essential, as management strategies differ drastically: EG requires immediate anti-pseudomonal antibiotics, necrotizing fasciitis mandates urgent surgery, and PG is treated with immunosuppressants. Here we report a neutropenic infant with fulminant community-acquired *P. aeruginosa* sepsis in whom early recognition of EG facilitated prompt multidisciplinary intervention.

## Case presentation

**Figure 1:**
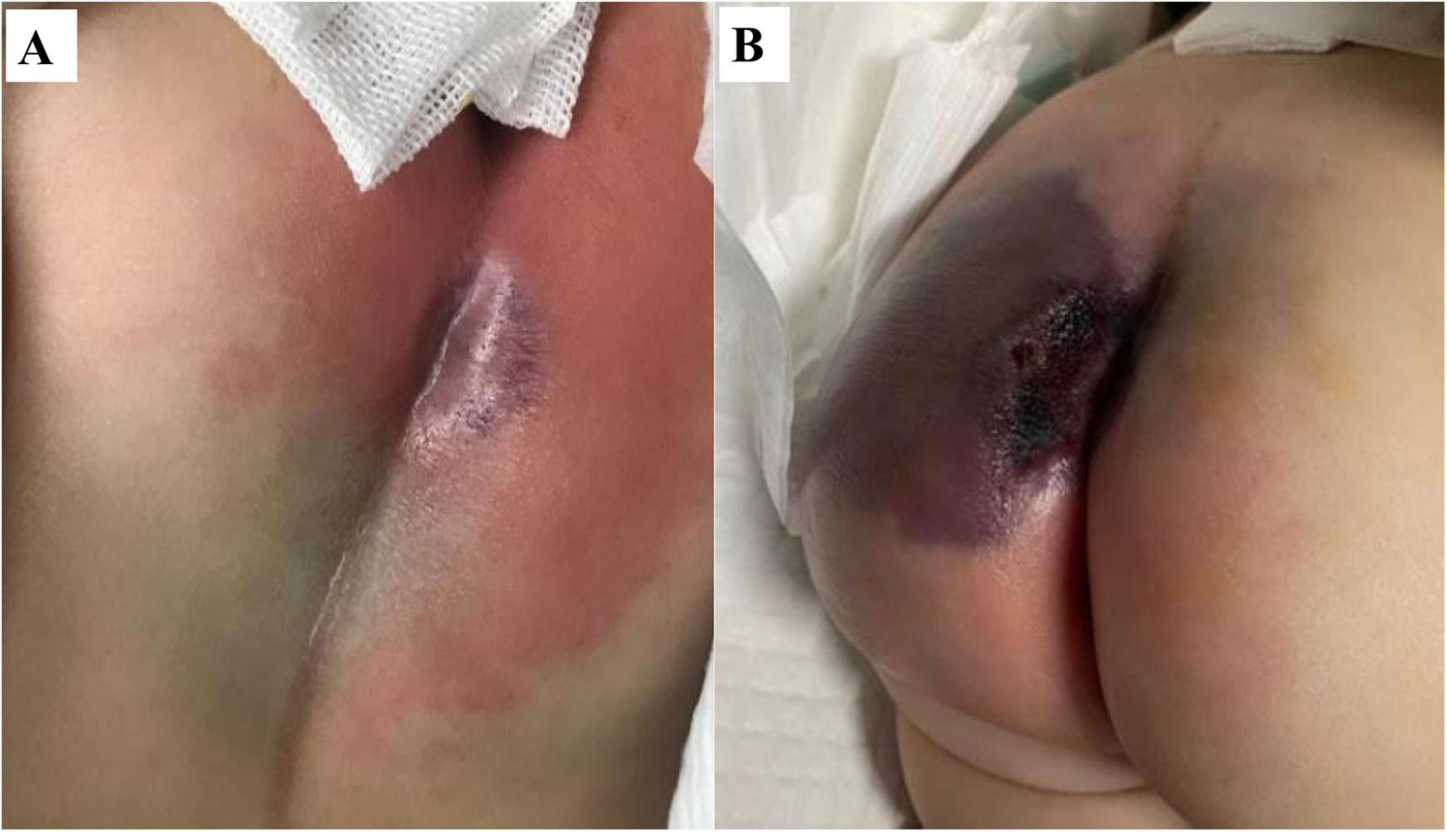
Rapid progression of ecthyma gangrenosum in a neutropenic infant with *Pseudomonas aeruginosa* sepsis. **(A)** Initial presentation: a well-demarcated violaceous plaque (1.5 cm × 1.5 cm) with surrounding erythema. **(B)** 24 h later: progression to a violaceous-black eschar with central necrosis and full-thickness ulceration.

A 4-month-old ex-preterm girl (gestational age 36 weeks) with a past history of neonatal jaundice was admitted with a 24-h history of high-grade fever (peak 39.5 ℃ recurring every 6 h), lethargy, and poor feeding. On admission, her vital signs indicated hemodynamic instability with hypotension (84/58 mmHg) and tachypnea (30 breaths/min). Physical examination revealed a violaceous indurated plaque in the perianal region (1.5 cm × 1.5 cm, Figure 1A) surrounded by erythema, highly suggestive of EG. The perianal lesion was not associated with significant pain upon initial examination. Initial laboratory investigations demonstrated profound neutropenia (absolute neutrophil count 0.08 × 10^9^/L), leukopenia (WBC 1.2 × 10^9^/L), markedly elevated inflammatory markers [C-reactive protein 146 mg/L; procalcitonin 11.57 ng/mL], and mild coagulopathy [prothrombin time 16.8 s, INR 1.48, activated partial thromboplastin time 51 s]. Cerebrospinal fluid analysis and immunological screening were unremarkable, excluding meningitis and primary immunodeficiency.

**Figure 2:**
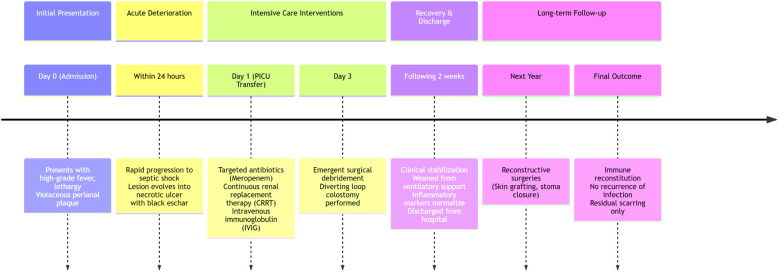
Timeline of clinical course, interventions, and outcomes.

**Figure 3:**
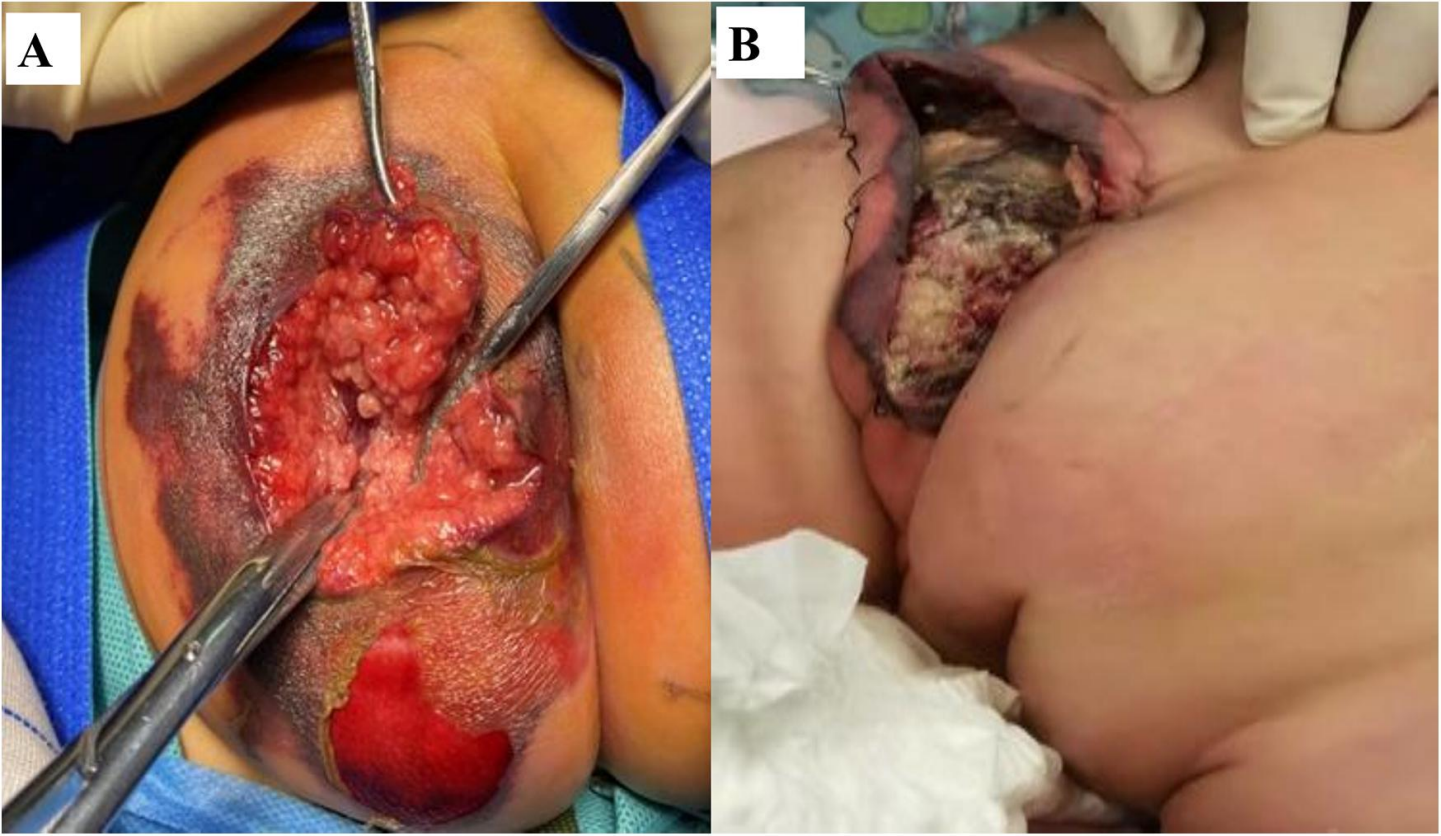
Surgical debridement and postoperative wound healing. **(A)** Intraoperative view showing extensive soft tissue necrosis in the gluteal region. **(B)** Postoperative follow-up demonstrating satisfactory wound healing.

Despite prompt initiation of empiric broad-spectrum antibiotics, the patient deteriorated rapidly within 24 h, progressing to septic shock with worsening hypotension and lesion evolution to a necrotic ulcer with black eschar formation (Figure 1B). This abrupt clinical decline necessitated transfer to the pediatric intensive care unit (PICU), where a multidisciplinary approach was urgently adopted. Susceptibility-guided anti-pseudomonal therapy with meropenem (20 mg/kg, q8h) was administered over a 15-day course, supplemented by additional agents such as cefoperazone/sulbactam and isepamicin to enhance coverage amid the patient's refractory course. Due to the lesion's rapid necrosis and systemic deterioration, urgent surgical debridement with diverting loop colostomy was performed on the third day of hospitalization—a critical intervention for source control. The management was further complicated by a cytokine storm, necessitating continuous renal replacement therapy and plasma exchange, and a deep vein thrombosis requiring anticoagulation due to the right lower extremity. Immunomodulation with intravenous immunoglobulin due to profound neutropenia and septic shock, and prolonged mechanical ventilation for nearly two weeks were integral to stabilization (Figure 2). Emergent surgical debridement of necrotic tissue was performed to achieve source control (Figure 3).

Blood and wound cultures subsequently confirmed *P. aeruginosa* infection, sensitive to multiple anti-pseudomonal agents. Histopathology of the surgical specimen showed epidermal detachment, dermal necrosis with a dense acute inflammatory infiltrate, and numerous bacilli, findings characteristic of ecthyma gangrenosum. We noted the absence of significant family history, genetic disorders, or psychosocial factors that could predispose to infection, based on further history-taking and the results of whole-exome sequencing which excluded an underlying primary immunodeficiency. Diagnostically, the case was challenging due to the patient's non-response to initial empiric therapy and the evolution of a paradoxical clinical course. The improvement in fever was offset by the onset of shock and the alarming progression of the perianal lesion, which advanced to full-thickness necrosis within hours. This critical juncture necessitated multidisciplinary reevaluation, culminating in the recognition of ecthyma gangrenosum as the underlying pathology and highlighting the challenge of diagnosing this condition amidst a rapidly unfolding septic picture. With combined antimicrobial therapy, immunomodulation, intensive organ support, and surgical management, the patient's condition gradually stabilized. Defervescence was achieved, inflammatory markers normalized, and the cutaneous lesions demonstrated progressive healing under regular wound care.

At discharge, the patient's hematological and immunological parameters had normalized. Following discharge, she underwent a series of reconstructive procedures, including skin grafting at a burn center and, the following year, a complex abdominal surgery to reverse the colostomy and address adhesions. On final follow-up, the patient exhibited no recurrence of infection and displayed normal immune function, with only residual scarring in the left gluteal and posterior thigh region as a sequela.

## Discussion and conclusions

This case highlights the fulminant nature of community-acquired *P. aeruginosa* sepsis in a previously healthy neutropenic infant. The progression from fever to hemodynamic collapse within 18 h illustrates the high virulence of this pathogen in immunocompromised hosts, consistent with reported mortality exceeding 30% in neutropenic patients (9, 10). The perianal manifestation of EG was a critical diagnostic clue of disseminated infection. EG represents hematogenous bacterial invasion of vascular media and adventitia, leading to thrombosis, ischemic necrosis, and cutaneous ulceration (11–13). Its rapid onset and violaceous border often precede systemic collapse, offering a narrow but vital diagnostic window. Key differential diagnoses include pyoderma gangrenosum, necrotizing fasciitis, and disseminated fungal infections.

Our case underscores the importance of differentiating EG from other rapidly progressive necrotizing conditions, particularly necrotizing fasciitis, as both can affect the perianal region and advance swiftly to tissue necrosis. The distinction, however, is critical as their management diverges fundamentally. A key differentiating feature is the characteristic pain: necrotizing fasciitis typically presents with severe, often disproportionate pain as an early hallmark due to rapid nerve involvement (10), whereas the initial lesion of EG is typically painless, as observed in our patient. Histopathological findings provide definitive differentiation. A punch biopsy in our case confirmed EG, revealing perivascular hemorrhage and neutrophilic infiltration with central necrosis (14). which reflects hematogenous bacterial invasion and vascular thrombosis. In contrast, necrotizing fasciitis is characterized histologically by widespread necrosis of the superficial fascia and subcutaneous tissue with obliterative vasculitis, a dense neutrophilic infiltrate, and positive Gram staining (10). Recognizing these distinct clinical and pathological features is essential to guide appropriate life-saving therapy—immediate surgical debridement for necrotizing fasciitis vs. urgent anti-pseudomonal antibiotics and targeted debridement for EG.

Therapeutically, this case underscores the dual challenge of host immunosuppression and rising antimicrobial resistance. While our isolate remained susceptible to conventional agents, surveillance from Shanghai has reported imipenem and meropenem resistance rates of 34.1% and 15.8%, respectively, among isolates from febrile neutropenic patients (3). This resistance profile frequently results from chromosomal mutation-mediated overexpression of efflux pumps, production of carbapenemases (particularly metallo-β-lactamases), and permeability defects (15). The profound neutropenia (ANC <0.1 × 10^9^/L) observed in our patient represents an independent predictor of mortality, necessitating aggressive supportive measures including granulocyte colony-stimulating factor administration and consideration of granulocyte transfusions in refractory cases (5, 15). Additionally, the emerging role of novel β-lactam/β-lactamase inhibitor combinations such as ceftolozane-tazobactam and ceftazidime-avibactam for multidrug-resistant strains warrants consideration in areas with high resistance prevalence.

Optimal management requires coordinated multidisciplinary intervention encompassing infectious diseases, critical care, pediatric surgery, and hematology expertise. Early surgical debridement remains paramount for source control, as devitalized tissue provides a sanctuary for bacterial proliferation and impedes antibiotic penetration (11, 14). Our experience reinforces that surgical consultation should be obtained immediately upon EG recognition, with urgent debridement of necrotic tissue significantly improving survival outcomes. The pharmacological approach must include combination therapy utilizing an anti-pseudomonal β-lactam with either an aminoglycoside or fluoroquinolone until susceptibility patterns are established, with subsequent de-escalation based on culture results. This case illustrates how prompt recognition of pathognomonic dermatological findings, combined with rapid initiation of targeted antimicrobial therapy, intensive supportive care, and surgical intervention, can significantly improve survival in invasive pseudomonal disease.

## Data Availability

The raw data supporting the conclusions of this article will be made available by the authors, without undue reservation.

## References

[1] Schmidt KD Tümmler B Römling U. Comparative genome mapping of *Pseudomonas aeruginosa* PAO with P. aeruginosa C, which belongs to a major clone in cystic fibrosis patients and aquatic habitats. J Bacteriol. (1996) 178(1):85–93. 10.1128/jb.178.1.85-93.1996 8550447 PMC177624

[2] Mielko KA Jabłoński SJ Milczewska J Sands D Łukaszewicz M Młynarz P. Metabolomic studies of *Pseudomonas aeruginosa*. World J Microbiol Biotechnol. (2019) 35(11):178. 10.1007/s11274-019-2739-1 31701321 PMC6838043

[3] Zhu J Hu J Mao Y Chen F Zhu J Shi J A multicenter, retrospective study of pathogenic bacteria distribution and drug resistance in febrile neutropenic patients with hematological diseases in Shanghai. Zhonghua Xue Ye Xue Za Zhi. (2017) 38(11):945–50. 10.3760/cma.j.issn.0253-2727.2017.11.009 29224317 PMC7342794

[4] Denic S Narchi H Al Mekaini LA Al-Hammadi S Al Jabri ON Souid AK. Prevalence of neutropenia in children by nationality. BMC Hematol. (2016) 16:15. 10.1186/s12878-016-0054-8 27213048 PMC4875641

[5] Solomou EE Salamaliki C Lagadinou M. How to make the right diagnosis in neutropenia. Clin Hematol Int. (2021) 3(2):41–6. 10.2991/chi.k.210216.001 34595466 PMC8432397

[6] Hurwitz RM. Ecthyma gangrenosum without bacteremia or necrotic cellulitis: a localized form of septic vasculitis. Arch Intern Med. (1987) 147(8):1513. Available online at: https://www.ncbi.nlm.nih.gov/pubmed/3632159 (Accessed September 10, 2025). 3632159

[7] Demirdover C Geyik A Vayvada H. Necrotising fasciitis or pyoderma gangrenosum: a fatal dilemma. Int Wound J. (2019) 16(6):1347–53. 10.1111/iwj.13196 31418533 PMC7948559

[8] Bellapianta JM Ljungquist K Tobin E Uhl R. Necrotizing fasciitis. J Am Acad Orthop Surg. (2009) 17(3):174–82. 10.5435/00124635-200903000-00006 19264710

[9] Huang YC Lin TY Wang CH. Community-acquired *Pseudomonas aeruginosa* sepsis in previously healthy infants and children: analysis of forty-three episodes. Pediatr Infect Dis J. (2002) 21(11):1049–52. 10.1097/00006454-200211000-00015 12442028

[10] Kang CI Kim SH Park WB Lee KD Kim HB Kim EC Clinical features and outcome of patients with community-acquired *Pseudomonas aeruginosa* bacteraemia. Clin Microbiol Infect. (2005) 11(5):415–8. 10.1111/j.1469-0691.2005.01102.x 15819873

[11] Shah M Crane JS. Ecthyma Gangrenosum. Treasure Island, FL: StatPearls Publishing (2025). (Disclosure: Jonathan Crane declares no relevant financial relationships with ineligible companies). 30521198

[12] Demircioğlu F Oren H. Ecthyma gangrenosum: sign of *Pseudomonas aeruginosa* bacteremia. Pediatr Hematol Oncol. (2008) 25(4):369–70. 10.1080/08880010802016102 18484484

[13] Chan YH Chong CY Puthucheary J Loh TF. Ecthyma gangrenosum: a manifestation of Pseudomonas sepsis in three paediatric patients. Singapore Med J. (2006) 47(12):1080–3. Available online at: https://www.sma.org.sg/smj/4712/4712cr1.pdf 17139406

[14] Vaiman M Lasarovitch T Heller L Lotan G. Ecthyma gangrenosum versus ecthyma-like lesions: should we separate these conditions? Acta Dermatovenerol Alp Pannonica Adriat. (2015) 24(4):69–72. 10.15570/actaapa.2015.18 26697730

[15] Garcia-Vidal C Cardozo-Espinola C Puerta-Alcalde P Marco F Tellez A Agüero D Risk factors for mortality in patients with acute leukemia and bloodstream infections in the era of multiresistance. PLoS One. (2018) 13(6):e0199531. 10.1371/journal.pone.0199531 29953464 PMC6023133

